# Total serum IgE levels and profile sensitization to dust mites in patients with asthma

**DOI:** 10.1186/1939-4551-8-S1-A140

**Published:** 2015-04-08

**Authors:** Rafael Teixeira Figueredo Poleshuck, Stephania Campregher Bertti, Norma Rubini, Albertina Varandas Capelo, Eliane Miranda Da Silva, Fernando Samuel Sion, Carlos Alberto Morais De Sa

**Affiliations:** 1 Federal University of the State of Rio De Janeiro, Brazil

## Background

Atopic asthma occurs in a significant percentage of patients in different age groups and the main sensitizing allergens are dust mites. The aim of this study was to assess the levels of total serum IgE, specific IgE to Dermatophagoides pteronyssinus (Dp) and Blomia tropicalis (Bt) in patients with asthma as well as the influence of gender, age and severity of disease in the total serum IgE levels and in the sensitization to mites.

## Methods

We conducted a retrospective, cross-sectional study with patients with asthma who were over 6 years of age and had regular follow-up. Data were collected from the Laboratory of Immunology records and database. The total serum IgE and specific IgE measurements were performed by ImmunoCAP.

## Results

Of the 79 patients analyzed, aging between 6 and 81 (mean = 35.78, SD = 53.08), 65% were female. IgE levels were elevated in 64% of patients, 54% had specific IgE sensitization to Dp and 52% to Bt. IgE levels were elevated in 73% of the children, 57% of the adolescents, 81% of the adults and 27% of the elderly. Statistical analysis was significant in the comparison between children and adults versus the elderly, p = 0.01 and p = 0.0001, respectively. The frequency of specific IgE sensitization was similar in both sexes and in the different age brackets (p> 0.05). The severity of asthma had no influence in the frequency of specific IgE sensitization to Dp and Bt (p> 0.05).

## Conclusions

We observed that the majority of asthma patients showed high IgE levels and half the patients had specific IgE sensitization to Dp and Bt. Our data indicate a lower frequency of high IgE levels in the elderly, pointing to a lower sensitivity of this method in the research of atopy in elderly asthmatics. There was no correlation between the levels of total serum IgE and specific IgE sensitization with asthma severity.

